# A proof-of-concept analysis of data from the first NHS clinic for young adults with comorbid cannabis use and psychotic disorders

**DOI:** 10.1192/bjo.2024.782

**Published:** 2024-12-12

**Authors:** Marta Di Forti, Benjamin W. Bond, Edoardo Spinazzola, Giulia Trotta, Jodie Lynn, Richard Malkin, Naba Kamran Siddiqui, Sultan Demir, Titilayomi Opadokun, Perry B.M. Leung, Zhikun Li, Andrea Quattrone, Gabriella Baxter, Elizabeth Appiah-Kusi, Tom P. Freeman, Hannah Walsh, Tommaso Squeri, Daria Semikina, Felicity Amberson-Jones, Isabelle Austin-Zimmerman, Tim Meynen, Diego Quattrone, Robin M. Murray

**Affiliations:** Department of Social Genetics and Developmental Psychiatry, Institute of Psychiatry, Psychology & Neuroscience, King's College London, UK; South London and Maudsley NHS Foundation Mental Health Trust, London, UK; Department of Psychosis Studies, Institute of Psychiatry, Psychology & Neuroscience, King's College London, UK; Tees, Esk and Wear Valleys NHS Foundation Trust, Darlington, UK; Department of Psychiatry, Li Ka Shing Faculty of Medicine, The University of Hong Kong, Hong Kong SAR; Centre for Neurodevelopmental Disorders New Hunt's House, Guy's Campus King's College London, UK; Institute of Biomedical Sciences Abel Salazar (ICBAS), University of Porto, Portugal; Addiction and Mental Health Group University of Bath, UK; Department of Addictions, Institute of Psychiatry, Psychology & Neuroscience, King's College London, UK; GKT School of Medical Education, King's College London, UK

**Keywords:** Cannabis use, psychosis, psychosocial interventions, personalised, cessation

## Abstract

**Background:**

Cannabis use severely affects the outcome of people with psychotic disorders, yet there is a lack of treatments. To address this, in 2019 the National Health Service (NHS) Cannabis Clinic for Psychosis (CCP) was developed to support adults suffering from psychosis to reduce and/or stop their cannabis use.

**Aims:**

Examine outcome data from the first 46 individuals to complete the CCP's intervention.

**Method:**

The sample (*N* = 46) consisted of adults (aged ≥ 18) with psychosis under the care of the South London and Maudsley NHS Foundation Trust, referred to the CCP between January 2020 and February 2023, who completed their intervention by September 2023. Clinical and functional measures were collected before (T0) and after (T1) the CCP intervention (one-to-one sessions and peer group attendance). Primary outcomes were changes in the Cannabis Use Disorders Identification Test-Revised (CUDIT-R) score and pattern of cannabis use. Secondary outcomes included T0–T1 changes in measures of delusions, paranoia, depression, anxiety and functioning.

**Results:**

A reduction in the mean CUDIT-R score was observed between T0 (mean difference = 17.10, 95% CI = 15.54–18.67) and T1, with 73.91% of participants achieving abstinence and 26.09% reducing the frequency and potency of their use. Significant improvements in all clinical and functional outcomes were observed, with 90.70% being in work or education at T1 compared with 8.70% at T0. The variance in CUDIT-R scores explained between 34 and 64% of the variance in our secondary measures.

**Conclusions:**

The CCP intervention is a feasible strategy to support cannabis use cessation/reduction and improve clinical and functional outcomes of people with psychotic disorders.

Cannabis use is potentially the most modifiable risk factor for psychotic disorders.^1,2^ Epidemiological, experimental and biological studies have indicated that heavy cannabis use increases the risk of psychosis phenotypes,^3^ from psychotic symptoms^4,5^ to purely clinical disorders, with a dose-response relationship.^6^ Moreover, cannabis use has been associated with an earlier age of illness onset^7,8^ and more severe positive symptoms.^9^

## Cannabis use and psychosis outcomes

Consistent evidence points towards the detrimental impact of continued cannabis use on both clinical and functional outcomes in those with psychotic disorders.^10,11^ Longitudinal follow-up studies of people with first-episode psychosis (FEP) indicate that continuing to use cannabis after illness onset is associated with shorter periods between relapses, more severe symptoms requiring hospital admission and longer bed stays.^10^ Indeed, a clinical records study from South London showed that those who continued to use cannabis over a 5-year follow-up were more likely to experience compulsory hospital admissions than those who ceased their use.^12^ Schoeler et al^13^ followed a group of FEP participants for 2 years and found that those who continued to use high-potency (skunk-like) cannabis every day had worse clinical outcomes than those who had stopped using cannabis after psychosis-onset.

Concern about the detrimental impact of continued high-potency cannabis use in people with psychosis has been compounded by the latest UK cannabis potency study, which reported that high-potency skunk-type cannabis had taken over most of the market, representing 96% of the cannabis used in London.^14^

## The Cannabis Clinic for Psychosis

Frequently, people with psychosis who use cannabis fall between general adult psychiatric services and those for addiction, with neither offering comprehensive therapy. Therefore, in 2019, thanks to an award from the Maudsley Charity, we developed the first National Health Service (NHS) Cannabis Clinic for Psychosis (CCP; maudsleycharity.org/case-studies/cannabis-clinic/), nested within the South London and Maudsley Foundation Trust (SLaM). The clinic aims to improve the outcomes for adults with psychotic disorders who use cannabis, by offering a comprehensive and flexible approach to cannabis use reduction/cessation. In this proof-of-concept paper, we present the outcome data from the first 46 individuals who completed their intervention with the CCP.

## Method

### Study design and participants

The sample comprised adults with psychotic disorders under the care of the SLaM who were referred to the CCP. This proof-of-concept analysis includes data from the people referred to the CCP's clinical service between January 2020 and February 2023, who completed their intervention by September 2023. Despite challenges posed by the COVID-19 pandemic, the CCP, funded by the Maudsley Charity, began accepting referrals in January. Initially, because of limited resources, referrals were only accepted from the Lambeth Early Onset (LEO) and Southwark Team for Early Psychosis (STEP) early intervention services (EIS) within SLaM. After February 2023, the CCP expanded to accept referrals from the whole of SLaM services. Eligibility criteria included adults aged 18 and above suffering from a psychotic disorder, under the care of SLaM and expressing the intention to explore changes to their current cannabis use. The CCP did not have funding to provide an interpreter; therefore, it could only offer the intervention to individuals able to speak and understand English.

Referrals came primarily from care coordinators (i.e. social workers, occupational therapists or nurses co-ordinating the patient community mental healthcare), medical staff and dual diagnosis practitioners. The CCP developed two leaflets, one for staff and one for patients, describing the intervention offered (see Supplementary Material available at https://doi.org/10.1192/bjo.2024.782). Referring staff were required to discuss the referrals with their patients and acquire their consent before emailing their details to the CCP staff (Supplementary Figure 1).

This manuscript analysis was carried out as part of a clinical service evaluation and did not require ethical approval.

### Intervention

The CCP intervention consisted of two main components: (a) one-to-one weekly sessions and (b) an online peer group run on Zoom every Tuesday at 16.00–17.00h.

The one-to-one sessions were initially run online only because of the COVID-19 pandemic; participants were later offered the choice of meeting online (Microsoft Teams/Zoom/WhatsApp), face to face or a hybrid of the two. Participants could change the meeting modality at any point to suit their needs (e.g. transport difficulties, family commitments or mental state). All were offered a minimum of 20 sessions, which did not have to be completed consecutively. The sessions lasted a maximum of 60 min, and each session duration was flexible and adjusted to meet people's needs. People were sent session reminders by either text, email or phone call the day before and on the day of their session. Individuals who co-used cannabis with tobacco were also referred to the smoking cessation team.

The one-to-one sessions were delivered by either a: (1) band 3/6 dual diagnosis practitioner, (2) trainee clinical psychologist or (3) a pair of MSc clinical placement students. All CCP staff received individual supervision from a consultant clinical psychologist and the CCP lead consultant psychiatrist. They also met once a week for a group clinical supervision with the lead consultant psychiatrist to review and reflect on the individuals’ progress and to plan the intervention.

During the one-to-one sessions, the CCP staff used a combination of evidence-based psychosocial interventions (PSIs), well established in the treatment of addictions, which, rather than following a pre-set protocol, were tailored to each person's needs while considering their psychosis comorbidity (Supplementary Table 1). Cognitive–behavioural therapy (CBT) and motivational interviewing were used, and where appropriate, imagery techniques were incorporated as a motivational amplifier (Supplementary Table 1).^15^

The weekly online peer group was facilitated by a senior member of the CCP staff and moderated by two peer mentors with lived experience of psychosis and cannabis use. Reminders with the leaflet containing the Zoom link and a description of each week's topic (see Supplementary Material) were sent via email or WhatsApp to patients and staff the week and day before, and on the day of the peer group.

Holding the peer group online allowed the CCP to host international experts, who gave talks on topics covering cannabis use and its effects, psychosis and addiction, followed by interactive discussions with those attending. The experts ranged from researchers, clinicians, authors and musicians to experts by experience. Participants could choose to keep their video off and use the chat to ask questions or share their experiences. During each peer group term, an external guest was invited to share their experience of recovering from psychosis after stopping their cannabis use.

### Measurements and materials

Before starting the intervention, the CCP core assessment was administered to each participant. This included validated questionnaires to collect sociodemographic data as well as data on cannabis use, psychopathology, mood, anxiety, cognition and level of functioning (see Supplementary Material). The core assessment was carried out both at baseline, the week before the first one-to-one session (T0) and after completion of the intervention (T1), within two weeks from the last session. Here we focus on the following data:

Sociodemographic: age, gender, ethnicity, relationship status, level of education achieved, level of social activity and employment status.^16^

Primary outcomes and their changes between T0 and T1:

1. Cannabis Use Disorders Identification Test-Revised score (CUDIT-R):^17^ an 8-item scale designed to screen for cannabis use disorder (X ≥ 9).^18^
2. Cannabis Experience Questionnaire modified version (CEQ-mv):^19^ used to assess cannabis use frequency and type of cannabis used.

Secondary outcomes and their changes between T0 and T1:

1. Psychotic Symptom Rating Scales Delusions Subscale (PSYRATS DEL):^20^ a 6-item scale designed to assess different dimensions of delusions, with higher scores indicating greater symptom severity.
2. State Social Paranoia Scale (SSPS):^21^ a 10-item measure of recent paranoid thinking in a social situation, with higher scores indicating greater levels of paranoia.
3. Generalised Anxiety Disorder-7 (GAD-7):^22^ a 7-item measure assessing recent levels of anxiety, with higher scores indicating greater levels of anxiety.
4. Patient Health Questionnaire-9 (PHQ-9):^23^ a 9-item measure that assesses recent levels of depression, with higher scores indicating greater levels of depression.
5. Global Assessment of Functioning (GAF)^24^ which attempts to establish an individual's recent level of functioning on a 1–100 scale, with higher scores indicative of superior functioning.
6. Changes in the level of social activities.

### Statistical analysis

Descriptive analyses for both categorical and continuous variables were run using R version 4.1.153 for Windows.^25^ Percentages were used to describe the sociodemographic categories, and continuous variables were summarised reporting the mean and s.d. and the median if non-continuously distributed. Differences in the primary and secondary outcome mean-scores (CUDIT-R, PSYRATS DEL, SSPS, GAD-7, PHQ-9 and GAF) and categorical variables (frequency of use, type of cannabis used and social activity) between T0 and T1 were calculated using within-samples *t*-tests or Pearson's chi^2^ and displayed using boxplots or bar charts.

First, we aimed to test if changes between T0 and T1 in our primary outcome scores covaried and were correlated with the secondary outcome scores. Using the change in our measures (T0–T1) in STATA 16 for Windows, first we tested for correlation (*pwcorr* command) between the CUDIT-R scores and the scores for each of the secondary outcomes (SSPS, PSYRATS DEL, GAF, GAD-7, PHQ-9), and built related scatter plots. Second, we measured how much of the variance in our secondary outcomes was explained by the variance in the primary outcome of the CUDIT-R, and we ran linear regressions adjusted for age, gender, ethnicity and baseline level of education to capture how much of the variance in each of the secondary outcome scores was explained by the variance in the primary outcome (CUDIT-R).

## Results

Between January 2020 and February 2023, the CCP received 131 referrals, 69.5% (*N* = 91) of them over the previous 8 months because of increased awareness of the service within the Trust and the employment of permanent CCP staff. A third of the initial referrals (*N* = 45/131, 34.4%) were not eligible (i.e. the referring staff were not yet clear about the eligibility criteria) and were diverted to other services (see details in Supplementary Figure 2); of the *N* = 86 eligible referrals 12.8%, (*N* = 11/86) stated they were not ready to engage, 9.3% (*N* = 8/86) dropped out after starting the intervention (see details in Supplementary Table 2), 14% (*N* = 12/86) were still receiving it, 10.5% (*N* = 9/86) were on the waiting list and 53.5% (*N* = 46/86) had completed the intervention by September 2023 as well as the core assessments at T0 and T1. On average the 46 people who completed the intervention received 20.9 (s.d. 1.9; median 20) one-to-one sessions over a period (T0–T1) of on average of 17 weeks (s.d. 4.6), each for an average duration of 40 min (s.d. 8.6).

### Sociodemographic and medication characteristics

Among the 46 people who completed the intervention, 40 (*N* = 40/46, 87%) were experiencing their first episode of psychosis and were under the care of SLaM EIS.

The remaining six were under the care of general community mental health teams, two were on long-acting first-generation depot antipsychotics and one on Aripiprazole depot, one on Paliperidone depot and the remaining two on once a day Risperidone oral, 6 mg and 8 mg respectively plus a selective serotonine reuptake inhibitor (SSRI). They had each experienced over four psychotic relapses associated with continuous use of cannabis.

All participants were compliant with their prescribed medication according to their clinical records and self-report. Among the first-episode cohort, 20/40 (50%) were prescribed Risperidone (mean dose = 4.5 mg, s.d. 1.05; median 4 mg), 4/40 (10%) were prescribed Quetiapine (mean dose = 287 mg, s.d. 103; median 300 mg), 10/40 (25%) were prescribed Olanzapine (mean dose = 15.75 mg, s.d. 5.00; median 17.50 mg) and 6/40 (15%) were prescribed Aripiprazole (mean dose = 9.16 mg, s.d. 1.29; median = 10 mg).

Of the overall sample, 14/46 (30.4%) had been on an antidepressants (SSRIs) for at least 2 months before their referral to the CCP. None of them were on mood-stabilisers or other classes of psychotropic medications between T0 and T1. For the whole sample, there were no changes in antipsychotic doses reported between T0 and T1, and only four of the individuals on antidepressants had a reduction of their SSRI dose before T1 (see Supplementary Material).

The overall group (*N* = 46) mean age was 25.67 (s.d. = 6.59). In all, 76.1% were male, 71.7% from ethnic minorities, 91% were single, 50% had left school with qualifications and 28.3% had progressed to further education, but 91.3% were socially inactive (Table 1).

**Table 1:** Summary of Cannabis Clinic for Psychosis (CCP) participant sociodemographic characteristics

	CCP patients (*N* = 46)	%
Gender		
Male	35	76.9
Female	11	23.9
Ethnicity		
Black	28	60.9
White	13	28.3
Mixed	5	10.9
Relationship status		
Single	42	91.3
Divorced/separated	2	4.4
In a relationship	2	4.4
Level of social activities		
Employed/in education	4	8.7
Social inactive	42	91.3
Highest educational attainment		
School, no qualifications	10	21.7
School, with qualifications	23	50.0
Tertiary, further	12	26.1
Higher (undergraduate)	1	2.2

### Pattern of cannabis use and clinical measures at T0

At the time of referral, 93.5% (43/46) of participants reported using high-potency (skunk-like) cannabis, 91.3% (42/46) used cannabis daily and the remaining 8.7% (4/46) used more than once a week; all but two smoked it with tobacco. The mean CUDIT-R score of this clinical group before starting the intervention was 18.15 (s.d. = 4.95), well above the threshold of 9 for CUD.

On average, the participants’ PSYRATS DEL score was 12.95 (s.d. = 6.47) and the SSPS was 62.10 (s.d. = 15.58). They presented with a GAD-7 mean score of 12.06 (s.d. = 7.65) and a PHQ-9 mean score of 11.56 (s.d. = 6.53), while on average the GAF score was 61.89, (s.d. = 8.56) indicating moderate symptoms and moderate difficulties in social occupation and general functioning.

### Change between T0 and T1

**Fig. 1:**
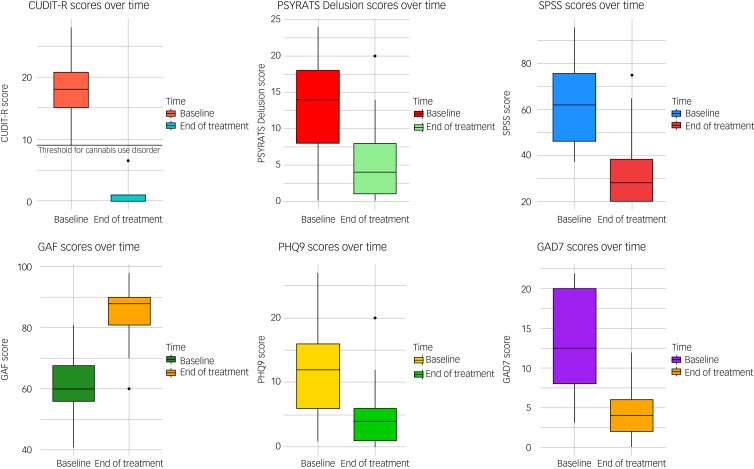
Box plots illustrating the changes between T0 and T1 in Cannabis Use Disorders Identification Test-Revised (CUDIT-R), Psychotic Symptom Rating Scales Delusions Subscale (PSYRATS DEL), Patient Health Questionnaire-9 (PHQ-9), Generalised Anxiety Disorder-7 (GAD-7) and State Social Paranoia Scale (SSPS) scores.

**Fig. 2:**
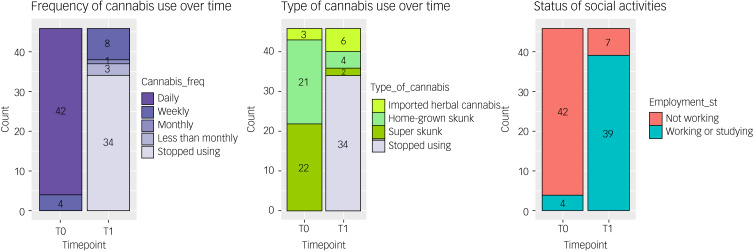
Bar chart illustrating changes in frequency of use, type of cannabis used and working/studying status between T0 and T1.

Between T0 and T1, we observed a change in the rates of cannabis use with 73.9% of participants (34/46) achieving full abstinence and the remainder significantly reducing their frequency of use (Pearson's chi^2^(4) = 81.33, *P* < 0.001). Among the 26.1% who continued to use cannabis, we found a significant shift away from high-potency (skunk-like) cannabis to low potency traditional herbal types (Pearson's chi^2^(3) = 58.50, *P* < 0.001). We also observed a significant reduction in the primary outcome measure and the mean CUDIT-R score (mean difference = 17.10, 95% CI = 15.54–18.67), as well as significant changes in the secondary outcome measures, reported in Table 2 and Fig. 1. Between T0 and T1 a significant proportion of the total sample became socially active with either working or studying (Pearson's chi^2^(1) = 53.48, *P* = 0.001) as shown in Fig. 2.

**Table 2:** Test of changes in continuous primary and secondary outcome measures at T0 and T1

	T0	T1	mD	95% CI	*t*	d.f.	*P*
CUDIT-R	18.15 (4.95)	1.04 (1.99)	17.10	15.54–18.67	21.72	90	<0.001
PSYRATS DEL	12.95 (6.47)	4.84 (4.57)	8.10	5.78–10.43	6.93	90	<0.001
GAD-7	13.28 (6.68)	4.08 (3.20)	9.19	7.02–11.36	8.40	90	<0.001
GAF	61.89 (8.56)	86.17 (8.27)	−24.28	−27.77–20.79	−13.83	90	<0.001
SSPS	62.10 (15.58)	31.00 (13.24)	31.10	25.11–37.10	10.31	90	<0.001
PHQ-9	11.56 (6.53)	4.26 (3.90)	7.30	5.07–9.53	6.50	90	<0.001

CUDIT-R, Cannabis Use Disorders Identification Test-Revised; PSYRATS DEL, Psychotic Symptom Rating Scales Delusions Subscale; GAD-7, Generalised Anxiety Disorder-7; GAF, Global Assessment of Functioning; SSPS, State Social Paranoia Scale; PHQ-9, Patient Health Questionnaire-9; mD, mean difference; *t*, *t*-statistic; *P*, *P*-value.

In addition, we found that the measured changes in secondary outcomes scores were correlated with the changes in CUDIT-R scores (SPSS *r* = 0.73; *P* < 0.00; PSYRATS DEL *r* = 0.58, *P* < 0.00; GAF *r* = −0.80, *P* < 0.00; GAD-7 *r* = 0.58, *P* < 0.00; PHQ-9 *r* = 0.61, *P* < 0.00; Supplementary Figure 3). Moreover, the variance in CUDIT-R scores, even when adjusting for age, gender, ethnicity and level of education, explained 54% of the variance in SSPS scores (*R*^2^ = 0.54; *P* < 0.00), 34% of the variance in PSYRATS DEL (*R*^2^ = 0.34; *P* < 0.00), 64% of the variance in GAF (*R*^2^ = 0.64, *P* < 0.00), 36% of the variance in GAD-7 (*R*^2^ = 0.36, *P* < 0.00) and 38% of the variance in PHQ-9 scores (*R*^2^ = 0.38, *P* < 0.00), which remained unchanged when we excluded the 14 individuals on antidepressants (Supplementary Figure 4).

## Discussion

### Main findings

The findings from this study support the feasibility of the intervention offered by the CCP to adults with psychotic disorders, who wish to reduce/stop their cannabis use. Indeed, 74% of the first 46 individuals who completed the CCP intervention stopped their use, with the remaining 26% reducing their use from daily to weekly or less, while using less potent cannabis types. These changes translated into a remarkable reduction in CUDIT-R scores, with the mean dropping to well below the threshold for Cannabis Use Disorder. Moreover, we observed an improvement in all the secondary outcomes with a particular and clinically relevant reduction in paranoia and delusion scores.^20,21^ The latter results are consistent with the existing literature which reports that cannabis use is mostly associated with positive psychotic symptoms and paranoia.^9,26^ Moreover, the reduction in depression and anxiety scores would be regarded as significant clinical improvements (see Supplementary Material); findings were not significantly different when comparing those people on antidepressants with the rest of the sample.^9,26^ Interestingly, we also observed an average increase in GAF scores of 20% following completion of the intervention (Fig. 2), which was validated by over 75% of the participants enrolling in education or starting a job. Finally, we were able to show that the changes in CUDIT-R scores between the baseline and end of treatment explained a significant proportion in the variance of the observed changes in paranoia, delusions, anxiety and depression, and a remarkable 64% in the level of functioning, even when adjusting for age, gender, ethnicity and level of education.

### Comparison with findings from previous studies

The clinical and social functional improvements reported following cessation/reduction in cannabis use are consistent with existing evidence. A recent 3–5-year follow-up study of individuals with established psychosis showed that cannabis cessation was associated with an improvement in psychotic symptoms as well as in level of functioning; in contrast, the latter further declined in those who continued to use cannabis.^11^ Furthermore, the relatively high level of patient social functioning we observed following cessation/reduction in cannabis use is consistent with data suggesting that people who develop psychosis in the context of cannabis use have better premorbid functioning than those who have never used cannabis.^27^

While psychological interventions seem to be effective in the treatment of cannabis dependence in those without other psychiatric comorbidities,^28,29^ randomised controlled trials testing interventions for cannabis cessation in adults with psychosis have not reported successful results.^30–33^ These studies often include psychoeducation and either one or a combination of two well-established psychological approaches for the treatment of addictions (e.g., motivational interviewing, CBT and contingency management).^30–33^ The number of sessions offered across these studies was predefined, followed a standardised protocol and had to be delivered within a specific time frame. Nevertheless, a study suggested that the duration of the intervention offered did not increase its efficacy when compared to treatment as usual.^32^ Moreover, pharmacological interventions, including antidepressants, antipsychotics or cannabinoid compounds aimed at reducing cannabis use in this group of people, have also provided only limited evidence of efficacy.^34^

Previous studies have suggested that the association between cannabis use and poor clinical outcomes of psychotic disorders is in part explained by poor medication adherence.^35,36^ Nevertheless, our data indicated that cannabis use reduction/cessation can positively impact prognosis, even in people who adhere to their pharmacological treatment for psychosis. Therefore, it is unlikely that our results can be explained by the pharmacological treatment prescribed, which remained unmodified for the duration of the time under the care of the CCP. Indeed, recent findings from the Optimization of Treatment and Management of Schizophrenia in Europe (OPTIMISE) study showed that individuals in remission from their first episode of psychosis were at higher risk of relapse if they used cannabis, regardless of whether they complied with their antipsychotic treatment or not. In that study, cannabis use clearly preceded relapse and poor cooperation as well as a decline in social functioning.^37^ Therefore, the development of an effective intervention to support this clinical population to stop/reduce their cannabis use has important secondary prevention implications, independently of medication adherence.

### Strengths and limitations

Our study findings need to be appraised in the context of limitations and strengths. First, the current analysis is based on an initial small sample size and includes predominantly young adults following their first psychotic episode, making our results less generalisable to the wider population of people with psychosis and cannabis use disorder. Second, we do not include a follow-up assessment, as the primary objective of this analysis was to test the feasibility of the CCP intervention and its efficacy on reducing cannabis use, not to measure the length of abstinence at follow-up.

Third, the CCP intervention was not evaluated against a control group. Therefore, the data we present should be considered as proof of concept of the feasibility of the CCP intervention rather than definite evidence of its efficacy.

Additionally, the information on cannabis use collected at T0 and T1 was not validated by biological measures, such as urine tests. Nevertheless, routinely available urine tests are only able to detect the presence or absence of cannabinoids and not changes in their amounts and therefore reduction in use or recent abstinence. Indeed, traces of cannabinoids can be detected in urine for up to a month following cessation of heavy use.^38^ While blood tests could be used to detect changes in levels of cannabinoids over time, they remain a costly option and are not available across all NHS laboratories. Furthermore, a consultation with our focus group of people with lived experience supported the reliability of self-reported use, as also indicated by previous research.^39–41^

However, an important strength and novelty of our study is the flexibility of the CCP intervention, which (a) implements multiple evidence-based PSIs, rather than focusing on just one or two, and (b) offers a flexible number of sessions, though within a replicable framework, of adjustable duration to respond to the person's needs, taking into consideration the impact on engagement and motivation of the comorbid psychosis. In this regard, a recent commentary on the clinical and cost-effectiveness of contingency management for cannabis use in early psychosis (CIRCLE) trial pointed out that a one-size-fits-all approach is unlikely to produce positive results given the complexity of the issue (e.g. different patterns of use and motivations for cannabis use should require a tailored approach^42^). Furthermore, we observed a drop-out rate of 9%, significantly lower than that generally reported for psychological intervention for individuals with dual diagnosis.^43^

Furthermore, the CCP team addresses from the start of participants’ engagement the impact of comorbid cannabis and tobacco use^44,45^ and works in close collaboration with the Trust smoking cessation services to offer each individual a tailored nicotine replacement treatment and related support.

A novel strength of the CCP intervention is its psychoeducation component which is delivered within the weekly peer group session by experts engaging in conversation with the patients/peers attending. The peer group offers the opportunity to learn about the latest research on cannabis and its uses, cannabis use disorder and psychosis, as well as offering a platform for discussion of shared experiences and peer-to-peer support during treatment.

### Qualitative data

Furthermore, the CCP feasibility is supported by the qualitative analysis we performed on data from the interviews of six individuals under the care of the service as well as eight healthcare professionals, who had referred people to the CCP. Indeed, the thematic analysis carried out indicated that the CCP: (1) improved awareness of the impact of cannabis use on psychosis, (2) changed perceptions of clinicians’ attitudes towards cannabis use, (3) facilitated the therapeutic relationship, (4) gave a central role to peers in recovery, (5) offered flexibility of service delivery, and (6) focused on motivations for change. Finally, patients and healthcare professionals identified multiple aspects of the CCP that facilitate access to care and thus meet the needs of individuals with psychotic disorder and comorbid CUD.

Here is a quotation from one of the participants who completed his treatment with the CCP in 2022:

*‘Now, with the clinic I am looking at life in a whole different way, my brain is starting to work again, I am doing an apprenticeship. It has changed my life.’*

More participants' quotations on the CCP are reported in the Supplementary Material.

### Implications

While future work is required to formally test the efficacy and cost-effectiveness of the CCP intervention, our findings support its feasibility and the importance of a flexible approach that combines addiction treatment interventions with psychosis treatment, alongside peer-centred psychoeducation models.

We do not advocate that the CCP is a model that every Trust in the country should embrace, but in catchment areas like the one of the South London and Maudsley NHS Trust, which has high incidence of psychosis and comorbid cannabis use disorder, the care of this clinical population cannot be delegated just to one professional per team without a coordinated treatment strategy, adequate supervision, support and training opportunities (see the quotation from health professionals interviewed on this topic in the Supplementary Material).

Importantly, our data evidence the reversibility of cannabis's negative impact on psychosis prognosis if use changes. Moreover, our findings strongly suggest that young adults suffering from psychotic disorders who stop or significantly reduce their pattern of cannabis use are more likely to become socially active. Therefore, at a time when changes in the legal status of cannabis might increase accessibility to cannabis and its potent types, it is important to develop services like the CCP that provide young adults with psychosis with the knowledge and support needed to prevent and/or reverse the negative impact that cannabis use can have on their illness course and their family, as well as mental health services.

## Supporting information

Di Forti et al. supplementary material Di Forti et al. supplementary material

## Data Availability

The data that support the findings of this study are part of a clinical service evaluation and therefore are available from the corresponding author M.D.F. upon reasonable request.
