# Supplementary material for: A proof-of-concept analysis of data from the first NHS clinic for young adults with comorbid cannabis use and psychotic disorders
Source: BJPsych Open. 2024 Dec 12;11(1):e1. doi: 10.1192/bjo.2024.782 (PMC11733490; doi:10.1192/bjo.2024.782)
Supplement: Di Forti et al. supplementary material [file S2056472424007828sup001.docx]

**Supplementary Material**

**S-Figure 1**. Diagram of the pathway from referral to end of treatment with the Cannabis Clinic for Psychosis (CCP) intervention.

| **S-Table 1.** Psychosocial interventions used during the CCP one-to-one sessions. | |
| --- | --- |
| **Intervention** | **Brief Description** |
| Cognitive-Behavioural Therapy | Exploring the thoughts, feelings, and beliefs that drive a patient's cannabis use behaviours, cravings, and triggers, as well as their connection with the patient’s symptoms. |
| Motivational Interviewing | Using a person-centred approach to guide and strengthen motivation to change. Imagery techniques such as guiding the development of images of helpful alternative behaviours and of previous past successes were used as a motivational amplifier. |
| Psychoeducation | Providing patients with insight into the effects that cannabis can have on all facets of physical and mental health, as well as harm reduction advice. |
| Contingency Management | The use of financial incentives (e-vouchers) to reward adherence to goals and incentivise further change. |
| SMART Goals | Assisting patients to set goals that are Specific, Measurable, Achievable, Relevant, and Timebound (SMART). |

**Details of the patients who dropped out:** The 8 patients who dropped out did not differ in their main socio-demographics nor in the medication prescribed to those who completed the intervention. They were male, single, in their early 20s (mean age=22, SD 2.41). Five of them were Black British and three White European, only two had completed school education and they were all socially inactive at time of referral. They were under the care of Early Intervention Services, therefore within their first episode of psychosis. They were all referred during the COVID-19 pandemic. Among these 8 patients, 6 had been prescribed Olanzapine (mean dose = mean dose=13.7 mgs, SD 5.4; median 12.5 mgs ) and 2 Risperidone, 4 mgs and 6 mgs respectively daily. None were on antidepressants or other psychotropic medications.

Their mean CUDIT-R score was 18 (SD=3.62), they used skunk-type cannabis daily, smoked with tobacco. Two of them also used methamphetamine intermittently and one was under the care of the alcohol and drug services for alcohol dependence. Compared to those who completed the intervention they had slightly higher anxiety (GAD-7), paranoia (SSPS) and PSYCHRATS -Del scores, and lower GAF scores (S-Table 2) than those who completed the intervention (see main text Table 2).

**S-Table 2:** Baseline measures of the 8 patients who dropped out

|  | **T0** |
| --- | --- |
| **CUDIT-R** | 18.1 (3.62) |
| **PSYRATS DEL** | 14.43 (2.95) |
| **GAD-7** | 15.67 (4.24) |
| **GAF** | 58.42 (3.26) |
| **SSPS** | 68.51 (4.81) |
| **PHQ-9** | 10.86 (3.52) |

Antidepressants T0-T1

Among the 46 patients who completed the intervention, 14 were on antidepressants, at T0 , 11 were on Sertraline (mean dose= 121.42, SD 66.11; median=150 mgs) and 3 on Citalopram 10 mgs. At T1, 4 of those who were taking Sertraline 200 mgs at T0, had a reduction of their dose to 100 mgs daily.

**S-Figure 3:** Scatter plots illustrating how the change scores (T0-T1) in SSPS (5a), PSYRATS Delusion (5b), GAD-7 (5c), PHQ(5d) and GAF (5e) relate to the change scores in CUDIT-R (N=46)

**S-Figure 4:** Scatter plots illustrating how the change scores (T0-T1) in SSPS (5a), PSYRATS Delusion (5b), GAD-7 (5c), PHQ(5d) and GAF (5e) relate to the change scores in CUDIT-R (N=46-14 patients on antidepressants)

**Discussion:**

The recorded changes in GAD7 and PHQ9 scores are regarding clinically meaningful as follows:

1. **Generalised Anxiety Disorder (GAD7) questionnaire (1).**

An IAPT scale

Number of items: 7

Scoring: 0-3

Total range: 0-21.

Caseness: The cut-off score is 8 and above

Reliable change score is 4 or more.

1. **Patient Health Questionnaire (PHQ)  (2)**

An IAPT ADSM

Number of items: 9

Scoring: Each item is scored between 0-3

Total range: 0-27

Interpretation: 0-4 (normal); 5-9 (mild depression), 10-14 (moderate depression), 15-19 moderately-severe depression and 20-27 (severe depression).

Caseness is a score of 10 or more.

Reliable change is defined as 6 or more

References:

**Reference**:

1. Spitzer RL et al (2006) A brief measure for assessing generalized

anxiety disorder: the GAD-7. Archives of Internal Medicine.166:1092-7 [https://doi.org/10.1001/archinte.166.10.1092](https://eur03.safelinks.protection.outlook.com/?url=https%3A%2F%2Fdoi.org%2F10.1001%2Farchinte.166.10.1092&data=05%7C02%7Ctim.meynen%40kcl.ac.uk%7C9e7647314df44598f60708dc1b4c5ecf%7C8370cf1416f34c16b83c724071654356%7C0%7C0%7C638415262021887752%7CUnknown%7CTWFpbGZsb3d8eyJWIjoiMC4wLjAwMDAiLCJQIjoiV2luMzIiLCJBTiI6Ik1haWwiLCJXVCI6Mn0%3D%7C3000%7C%7C%7C&sdata=J7Y5LmavN7dF%2BczBaZE5wJlTZVZhrF6MqKWF9bJe4Co%3D&reserved=0)

1. Kroenke K, et al (2001) The PHQ-9: validity of a brief depression severity measure. Journal of General Internal Medicine. 16:606-13. [https://doi.org/10.1046/j.1525-1497.2001.016009606.x](https://eur03.safelinks.protection.outlook.com/?url=https%3A%2F%2Fdoi.org%2F10.1046%2Fj.1525-1497.2001.016009606.x&data=05%7C02%7Ctim.meynen%40kcl.ac.uk%7C9e7647314df44598f60708dc1b4c5ecf%7C8370cf1416f34c16b83c724071654356%7C0%7C0%7C638415262021873832%7CUnknown%7CTWFpbGZsb3d8eyJWIjoiMC4wLjAwMDAiLCJQIjoiV2luMzIiLCJBTiI6Ik1haWwiLCJXVCI6Mn0%3D%7C3000%7C%7C%7C&sdata=e%2Btwv%2B9m%2B%2FhRSolDIB1blM%2BGVX0pg3%2FNh4WwKE5ofAQ%3D&reserved=0)

**CCP patients’ quotes** from the qualitative study carried out by Miss Gabriella Baxter, co-author of this paper:

- Here specific feedback on the PEER group from one of the patients attending regularly:

“People feel like their views are respected and people have an opportunity to speak in a safe

space and people feel listened to. People are encouraged to interact, when they can, where

they're able to, no one's forced to do anything.”

“So they've got their people in the group that have become abstinent. And that's really good

for the motivation.”

- The categorical outcome measures on level of functioning, which report on “ being or not socially active” are from patients and reflect the practical changes to their level of functioning. We also have the following quotes from the qualitative study:

“They've supported me in applying for a course, to try volunteering and just like, you know now I am doing it.

And so, yeah, they have been a great help.”

“They helped me with not just smoking cannabis. They supported me with finance, setting up a savings account, they supported me with my job application and read over my application and cover letter, they made a PowerPoint to show me my progress and what kind of changes i’m making. I now have a job”

- More general quotes on the one to one sessions:

“Everyone's like really welcoming, it feels like more of a friendly environment rather than like

a sit down and listen to what I'm saying environment”.

“I thought it would be stricter” and “I thought they would like tell me off

if I didn’t quit straight away but it’s very reasonable, I wouldn't have been able to quit without the clinic”.

“not judgmental, you know, when I started that, they didn't say, all right, stop smoking now. You know, they reduced it, reduced it in a way where we reduced it, reduced it, and then the Sativex.”

“What made me feel even more comfortable was they were giving me emotional support at

the same time and were motivating me and encouraging me to achieve more and aim higher

and that made me want to work on it even more.”

**Health professionals**’ **quotes** part of the Qualitative analysis carried out by Miss Gabriella Baxter, in support of the service, which stated: 1. “I think one of the big problems is the desperate shortage of time with care coordinators who have been told to do various other things.” 2. “The challenges around the time to come to training and how you best promote and train staff.
